# Sustainable Smart Cities—Social Media Platforms and Their Role in Community Neighborhood Resilience—A Systematic Review

**DOI:** 10.3390/ijerph20186720

**Published:** 2023-09-06

**Authors:** Soumya Balakrishnan, Suzanne Elayan, Martin Sykora, Marin Solter, Rob Feick, Christopher Hewitt, Yi Qiao Liu, Ketan Shankardass

**Affiliations:** 1 Centre for Information Management, Loughborough Business School, Loughborough University, Loughborough LE11 3TU, UKm.d.sykora@lboro.ac.uk (M.S.);; 2 School of Planning, University of Waterloo, Ring Rd, Waterloo, ON N2L 3G1, Canada; 3 Department of Health Sciences, Wilfrid Laurier University, Waterloo, ON M5B 1W8, Canadakshankardass@wlu.ca (K.S.)

**Keywords:** community resilience, social media, smart cities, COVID-19

## Abstract

The COVID-19 pandemic took most communities off guard and has highlighted gaps in community preparedness and resilience in spite of the numerous technological advancements and the variety of available social media platforms that many relied on during lockdown periods. This served to emphasise the necessity for exploring the roles of social media and smart city technologies in mitigating pandemic impacts. In this systematic literature review, we examined twelve articles on social media usage and smart city technologies and their contributions to community resilience during COVID-19. The analysis focused on the use of social media platforms and smart city technologies during and after lockdown periods, examining their role in fostering community resilience. Results indicate that social media and smart city technologies were instrumental in helping communities adapt and recover from the pandemic. While past studies have examined community resilience, social media, or smart cities separately, there is limited literature collating insights on the three elements combined. We therefore argue that these technologies, employed collaboratively, enhance community resilience during crises. Nevertheless, further research is recommended, particularly on urban resilience and comparative analyses to deepen our understanding of the complex interplay between these variables.

## 1. Introduction and Background

On 30 January 2020, the coronavirus outbreak was termed as an “International Concern” [1] related to a public health emergency by the World Health Organization (WHO). By 3 March 2020, the outbreak escalated to unprecedented levels which led to the WHO later declaring it as a global pandemic [1].

With the pandemic, the global population was not only confronted with the real threat to health from the virus, but also economic, social [2], and mental health [3] challenges. It also introduced the global population to what was dubbed by many as a “new normal”, where the pre-existing capabilities of technologies had to be readapted to newer ways of working [4]. Technology became essential in helping communities and individuals adapt, cope, recover, or stay connected with their friends and family throughout the pandemic lockdown periods [5]. Social media in particular influenced the global population’s adaptation and pandemic responses [6]. The dissemination of information and responding to situations, either in a positive manner or a negative manner, was quick and efficient [7].

While information sharing through social media led to the pandemic being described as an “infodemic” [8], social media has played a role in the mobilization of economic and social resources, providing support and help to communities and individuals, aiding in psychological and physical wellbeing. Social media contributed to community resilience, despite the negative discussion on how social media contributed to misinformation, fake news, social and mental triggers, etc. [9].

The COVID-19 pandemic has exposed the vulnerabilities of global cities and communities to unexpected challenges, such as public health crises [10]. Despite technology’s pervasive role in our lives and its contributions to innovation and social unity, it was evident that communities were unprepared for a crisis like the COVID-19 pandemic. This highlights the urgent need for a sustainable community resilience model that effectively leverages technology, especially in the face of growing urban populations [11], especially in order to manage major challenges [12].

As such, this study intends to bridge the gap in current research by examining the role of social media platforms during lockdown and post-lockdown in building community resilience as well as by exploring the impact of smart city initiatives on pandemic recovery and community resilience. This systematic literature review endeavors to assess prior research and establish a holistic understanding of these areas. 

Our study aims to examine the use of social media and smart technologies during the COVID-19 crisis and their influence on community resilience. The focus is twofold: analyzing social media’s role during and after lockdown and evaluating technology’s part in bolstering resilience in sustainable smart cities. A systematic literature review was undertaken, and despite existing research on social media’s impact, this study strives to fill gaps by assembling the relevant literature, thus laying the groundwork for future research in this area.

The main objectives of this study are:

- To understand how social media was used during lockdown periods to facilitate community resilience.
- To explore the use of social media post-lockdown for community resilience.
- To evaluate the impact of smart city initiatives on community resilience.

In light of the vulnerabilities exposed by the COVID-19 pandemic, there is an increasing realization of the need for sustainable resilience models in global cities. While technology, particularly social media, has profoundly influenced our daily lives, its potential to strengthen community resilience during crises remains underexplored. This study, therefore, aims to delve deeper into the role of social media and smart technologies during the pandemic, focusing on their implications for community resilience. By conducting a systematic literature review, we hope to address existing research gaps such as the lack of studies examining holistically the role social media and other existing smart city technologies could play in enhancing or hindering community resilience during crises as these have not yet been comprehensively explored or understood, and we therefore endeavor to lay a solid foundation for future investigations.

### 1.1. Community Resilience

Community resilience is a conceptually broad term that spans an array of disciplines used to describe a variety of approaches, ranging from grassroots groups to formal institutions [13]. It is exactly this multitudinous nature of the term that makes it so valuable, as it is useful for understanding and describing processes at varying levels of analysis. Fundamentally, community resilience can be understood as a process where community resources are developed and engaged with the aim of “responding to and influencing change, sustaining and renewing the community, and developing new trajectories for the communities’ future” [14].

Community resilience is crucial in understanding how successful collective organization can ensure wellness, functioning, and quality of life for a community that is confronted with challenges. Effectively, a measure of resilience is the capacity to which a community can reduce risk and resource inequities, develop productive connections, protect essential needs, and constructively address the crisis they are facing [13]. Fenxia [15] explored community resilience during the pandemic and identified a positive correlation between a community’s preventive measures against the pandemic and their resilience scores. They found that active communities in COVID-19 prevention had higher resilience. Notably, effective information dissemination through various media, like the internet and television, was a top factor highlighted by respondents. Despite these findings, Fenxia [15] emphasized the pressing need for a community-centric resilience framework. The need to assess community resilience in general was acknowledged by Cohen et al. [16], and a critical review conducted by Sharifi [17] identified several frameworks for assessing community resilience on an urban level. After examining several tools designed to assess community resilience, they found that most of the tools identified in their study primarily focus on evaluating a community’s capacity to recover from crises. For a comprehensive list of tools that measure and assess community resilience, please see Sharifi [17]. As for the specific concept of pandemic community resilience, Suleimany et al. [18] conducted a systematic literature review on community pandemic resilience, and they define it as the collective capacity of a community to anticipate, prepare for, respond to, and recover from pandemic events.

The COVID-19 pandemic forced communities to re-evaluate the extent of their resilience and modify their existing systems for a post-COVID “new normal” [19], revealing their vulnerability to unforeseen crises [10], and the urgency of community resilience and the need of its further development was clearly identified [20]. While Chandra et al. [21] find that community resilience can be achieved through combining organizations, social connections, and communication, Yip et al. [20] emphasize the need to incorporate civic-mindedness and social responsibility to foster unity during challenging times in order to expedite recovery.

### 1.2. Social Media and Community Resilience during Lockdown

Throughout the lockdown period, the reliance on social media saw massive increases globally [22]. It was integrated into sectors and areas where one would least expect; the UK government, for example, used WhatsApp to strategize during lockdown [23]. Another example is the use of WhatsApp and YouTube for e-learning purposes during lockdown in Jordan, where instructors would share their lectures with their students via social media [24]. The Slovak National Library found itself closed to the public; consequently, its workers used social media channels to work together to launch a free digital library for Slovak citizens [25]. In Nigeria, social media was used to enable churchgoers to worship from their homes [26]. China saw a massive increase in fitness exercise videos on TikTok [27]. There are numerous examples; as different locations used social media in resilient and innovative ways to combat the isolation created through lockdown restrictions, it undertook several roles and became a medium for information exchange, a method of offering and seeking support, and a coping mechanism for the emotional wellbeing of individuals, thus aiding in community resilience [28]. Sun et al. [29] conducted a 30-day diary study investigating the relationship between daily technology-related communication, user activity status, and wellbeing. The findings were mixed. Consistently, face-to-face interactions had a positive influence on wellbeing, suggesting that physical presence maintains its value even during lockdowns. Interestingly, the study revealed a positive correlation between active social media engagement and wellbeing. However, this correlation was not found among passive social media users, indicating that the level of interaction on these platforms can significantly influence an individual’s ability to respond to and recover from substantial challenges. Rolandi et al. [30] had similar positive observations in a study that focused on vulnerable older adults. It was found that vulnerable older adults who used social media felt less isolated during lockdown as they could maintain social connections even during difficult times.

### 1.3. Social Media and Community Resilience after COVID-19 Lockdowns

The lockdowns that communities experienced were life-altering [31], and the reliance on social media has grown as a result. Platforms such as Tiktok, Pinterest, Reddit, etc., grew from 2019 to 2021 to about 38%, 32%, and 30%, respectively [32]. While the role of social media continues to be debated, the role it plays post-lockdown and whether it is continuing to contribute to building community resilience post-lockdown remains unexplored.

Saghin et al. [33] conducted a study on social cohesion and community resilience, finding that social platforms were effective in the mobilization of resources in times of need, from collecting and distributing food to coordinating activities to crowdfunding, among others. Xie, Pinto, and Zhong [34] used gratification theory and coping theory in an attempt to understand the role of social media in post-lockdown community resilience. They argue that whether or not individuals perceive the resilience of their communities is a crucial element of the recovery process.

### 1.4. Sustainable Smart Cities

According Anthopoulos [35], a smart city is an urban area that uses electronic methods and sensors to collect data with the aim of improving operations and efficiencies across the city. This includes data collected from citizens, devices, buildings, and assets that is then processed and analyzed to monitor and manage urban resources, infrastructure and other community services to improve the quality of life of its population and creating resilient and empowered communities. Arguably, social media data can also contribute towards a smart city [36].

The principle of smart cities has gained increasing attention in recent scholarship, particularly during the COVID-19 pandemic, as highlighted by Sharifi, Khavarian-Garmsir, and Kummitha [37], while it has also received criticism for its technocentric and technocratic emphasis [38]. However, the subfield of sustainable smart cities, where environmental sustainability and technological advancement intertwine, remains relatively underexplored. It is predicted that by 2050, urban populations will encompass 66% of the world’s total, a significant increase from the current 54% [39]. This growth highlights the necessity for cities to strategically manage their resources to accommodate this population surge.

A smart city integrates information and communication technology into its infrastructural and operational framework [39]. Yet, Evergreen [40] argues that the term ‘smart city’ is often deployed loosely, with inadequate consideration of whether the outcomes or processes truly embody the ‘smart’ label. However, Unece [41] argues that the concept of a sustainable smart city interweaves the sustainability dimension into the utilization of technology. It highlights the importance of considering both current and future generations across diverse domains (such as social, economic, cultural, environmental, etc.). In this perspective, smart cities are envisaged as resilient, inclusive urban spaces that leverage technology to enhance the quality of life for all residents [39].

In light of the COVID-19 pandemic, it was observed that urban areas, with their dense population, bore the brunt of the impact [42]. The role of technology in these urban spaces, however, proved instrumental in managing the virus’s spread [43]. This emphasizes the importance of sustainable practices and the need for increased investment in sustainability [44]. Thus, the pivot towards more resilient and sustainable smart cities necessitates a stronger focus on integrating technological initiatives within the broader urban spatial context. There is limited existing research specifically on how smart city initiatives directly foster community resilience during times of health crises, and we therefore are providing this systematic literature review as a comprehensive aggregation and analysis of existing studies on this topic, aiding researchers in identifying best practices or lessons learned.

## 2. Methods

We conducted a systematic review to understand the role of social media in community resilience during the COVID-19 pandemic (during lockdown and post-lockdown periods). Systematic literature reviews are evidence-centered and aimed to answer specific research questions by identifying, screening, and selecting the literature that supports the objectives of the study [45], which in turn aids in recognizing further research opportunities [46]. We adopt the Preferred Reporting Items for Systematic Review and Meta-Analysis (PRISMA) statement for effective reporting, transparency, and reproducibility. The PRISMA statement also affords a clear description of the motivation, the processes followed, and the observations [46].

As a part of the literature review, the historical literature and current literature pertaining to the usage of social media platforms during the pandemic, more specifically the lockdown and post-lockdown period, were analyzed with a focus on studies related to building community resilience particularly in sustainable smart cities. To compile studies which are relevant to our research objectives, inclusion and exclusion criteria were defined—these will be discussed in depth in the following section.

### 2.1. Data Collection Methods

The systematic literature review began with the determination of inclusion and exclusion criteria of the study as it defines the basis for the research. This was followed by the identification of search keywords, search strategy, narrowing down the databases for study, and article screening. The details of each step involved in the data collection process are described in the following sections.

#### 2.1.1. Inclusion Criteria

To include literature that complimented the research objectives, inclusion and exclusion criteria were defined for the study based on the research objectives [47]. The inclusion and exclusion criteria of the study were as follows:

*Timeframe*: The current study considered only the literature pertaining to research conducted during the lockdown and post-lockdown period of the COVID-19 pandemic. Thus, studies published between November 2019 and August 2022 were considered as eligible for being a part of the systematic literature review. November 2019 was considered as the start time of the study as the first origin of corona virus was reported during that time.

*Primary Focus:* Our primary focus, based on our research objectives, considered studies for our systematic literature review that were as follows:

- Studies that explored the use of social media during lockdown and post-lockdown throughout the pandemic in the light of community resilience.
- Studies pertaining technologies that contribute towards a sustainable smart city, thus making urban spaces and their communities resilient to adverse events.
- Social media for the purpose of this study encompasses any online digital space that facilitates the sharing of user-generated content (such as Facebook, Instagram, TikTok, Twitter, YouTube, WhatsApp, etc.).

*Geographical Location*: The study was not bound by geographical borders and therefore includes any relevant literature available from studies conducted worldwide.

*Language*: Due to language constraints, only published articles in English which had full text availability were considered for this study; however, we will endeavor to include other languages in further studies.

*Databases*: We relied on three reputable databases, namely, Scopus, Web of Science, and EBSCO. Scopus was chosen for the study due to its extensive content availability (60.3% active journals), hassle-free user interface, and advanced search functionality [48]. Similarly, Web of Science was selected due to its high-quality content and access to over 12 million full-text articles [49]. EBSCO provided the intuitive platform for research availability of quality content and refined search features, thus making it a preferred choice for our systematic literature review (EBSCOhost research platform, 2022).

#### 2.1.2. Exclusion Criteria

The exclusion criteria for the studies were as follows:

- Duplicate studies;
- Magazines or letters;
- Studies which were not related to social media or smart cities;
- Studies which did not have full text availability;
- Studies in languages other than English.

### 2.2. Strategy for Identifying Literature

Our search strategy was developed using a PICO (population, intervention, comparison, and outcome) worksheet. PICO is a framework that is used to develop research questions as it helps in portraying all the essential components for a focused question [50]. The research questions framed for the study were as follows:

- Research Question 1: How was social media (I) used during COVID-19 lockdown (C) in contributing towards pandemic resilience (O) amongst the community (P)
- Research Question 2: How was social media (I) used after COVID-19 lockdown (C) in contributing towards pandemic resilience (O) amongst the community (P)
- Research Question 3: Did smart technologies (I) play a role in community resilience (O) in smart cities (P) during the COVID-19 lockdown (C)

Research questions 1 and 2 were combined under one PICO worksheet and research question 3 was separately compiled.

The search queries for each database along with the number of search results for each are provided below:

Research Questions 1 and 2—Prior to finalizing the keywords for the actual search, several mock searches were conducted in the databases to examine the clarity, diversity, and volume of the available literature. Search results were tested using terms like ‘urban’, ‘urban communities’, ‘social networks’, ‘shutdown’, ‘border closure’, ‘quarantine’, ‘curfew’, ‘urban resilience’, ‘pandemic response’, and ‘neighbourhood resilience’. The mentioned terms were used in combination with keywords which were a part of the final search during the trial search phase. However, the search results using the above stated terminologies were either too large, too sparse, or not relevant to the objectives of this study, and hence the searches had to be invalidated.

The finalized keywords were chosen from among the listed keywords based on the most popularly used terms in the reporting of social media in the context of the pandemic. The same keywords were used across the three databases to maintain consistency.

Web of Science—In the search conducted on Web of Science, 148 results were shown when the search was conducted for titles with the given keywords. A title search was conducted in Web of Science as it did not provide the option of searching using titles and abstracts in one search.

(((((((((((TI = (social media platforms)) OR TI = (facebook)) OR TI = (twitter)) OR TI = (social networking sites)) OR TI = (social networking websites)) OR TI = (social media) OR TI = (SNS)) AND TI = (society)) OR TI = (community)) OR TI = (neighborhood)) AND TI = (during lockdown)) OR TI = (post lockdown) OR TI = (lockdown) AND TI = (resilience) AND TI = (social media usage))

Scopus—The search was conducted on Scopus applying the same keywords used in Web of Science. Unlike Web of Science, Scopus provided the option to do title and abstract searches. The search produced 26 results.

TITLE-ABS ((((social AND media AND platforms) OR (facebook) OR (twitter) OR (social AND networking AND websites) OR (social AND media) OR (sns)) AND ((society) OR (community)) OR (neighborhood)) AND ((during AND lockdown) OR (post AND lockdown) OR (lockdown)) AND ((resilience) OR (social AND media AND usage)))

EBSCO—A total of 24 results were shown when the same search keywords were used in EBSCO for all text searches. The results were further filtered for studies from 2020 to 2022 which brought down the search results to 23. For the start year, 2020 was chosen, as the month filter option was not available in EBSCO.

TX (social media platforms or facebook or twitter or social networking sites or social media or sns) AND TX (during lockdown or post lockdown or lockdown) AND TX resilience AND TX social media usage AND TX (society or community or neighborhood)

Research Question 3—Prior to the actual search, a mock search was conducted on the databases using terms like ‘urban resilience during COVID-19′, and ‘urban resilience’; however, these terms had to excluded from the final search as the results were minimal. The final search was conducted using the same terms across all the three databases. The queries and description are provided below:

Web of Science—In the initial search with the keywords harvested, a total of 1682 results were generated by the database; however, most of the results were not relevant to the concept of the “smart city”. Hence, an additional search was conducted within the results with the keyword “smart city” which brought down the search results to 6. The keyword search was conducted for the title search in Web of Science.

(((((((((((TI = (sustainable smart cities)) OR TI = (smart city)) OR TI = (digital city)) OR TI = (intelligent city)) AND TI = (icts)) OR TI = (information and communication technologies)) OR TI = (during lockdown)) OR TI = (post lockdown)) OR TI = (lockdown)) OR TI = (COVID-19)) AND TI = (resilience))

Scopus—While using the search keywords for Research Question 3, a total of 4 results were generated. Like research Questions 1 and 2, the search was run for title and abstract in Scopus.

TITLE-ABS ((sustainable AND smart AND cities) OR (smart AND cities) OR (digital AND city) OR (intelligent AND city) AND ((icts) OR (information AND communications AND technology)) AND ((during AND lockdown) OR (post AND lockdown) OR (lockdown) OR (COVID-19)) AND ((resilience)))

EBSCO—An all-text search in EBSCO for smart city related search produced only 1 result. This could be indicative of lower research availability on this topic.

TI (sustainable smart cities or smart city or digital city or intelligent city) AND (icts or information & communication technologies) AND (during lockdown or post-lockdown or lockdown or COVID-19) AND resilience.

#### 2.2.1. Harvesting Search Terms

The search terms were then formulated based on each of the key elements in the PICO framework. Based on PICO, search terms for population, intervention, comparison, and outcome were harvested with the help of database tools like thesauri and the Oxford English Dictionary to come up with synonyms for the search terms. Search terms for the research questions are represented through Table 1 and Table 2.

#### 2.2.2. Searching the Databases

For the systematic literature review, data collection was dependent on keyword and inclusion-criteria-based searches of the chosen databases (Scopus, Web of Science, and EBSCO).

Like PICO, Research Questions 1 and 2 were combined into one search and Research Question 3 was searched separately. The same keywords were used in the three databases; however, the number of search results varied in each.

The process of screening the search results involved the extraction of papers and assessing their titles, abstracts, and objectives before deciding whether these were eligible for further screening. The second level involved full text review for identifying the most relevant studies for the research. The review process will be discussed at length in the Results section.

## 3. Results

### 3.1. Screening Results

A total number of 209 articles were analyzed during screening. This constitutes 198 articles related to social media use and 11 articles related to understanding the role of smart technologies in building resilience in smart cities. The PRISMA diagram given under the screening section summarizes the details of the screening process with a clear segregation of both social-media-usage-related searches and sustainable-smart-city-related searches. It was observed from the searches that social-media-related resilience is a widely explored research area, whereas sustainable-smart-city-related resilience, particularly through the lens of the pandemic, is in need of more research, particularly since many scholars have argued that sustainable smart cities can contribute to community resilience, particularly in trying times [43].

The literature screening process was facilitated by Rayyan, software specifically designed for systematic reviews (we used the online free version—see https://www.rayyan.ai/ accessed on 15 August 2022). Search outcomes from the databases were exported in RIS file format and subsequently imported into Rayyan. The results, comprising titles and abstracts of 209 studies, also contained all essential study-related information (such as authors, publication dates, etc.). To enhance the review’s clarity and efficiency, the process was divided into two distinct reviews: one focusing on community resilience and social media usage, and the other on smart cities and community resilience. The screening process was conducted in two stages: the identification stage and the screening stage.

- The Identification Stage

In the identification stage of the studies, 4 articles were excluded, as those were duplicate records which brought down the number of articles to 205 eligible ones for screening. The duplicate records were identified via Rayyan, which helped eliminate such articles prior to the screening process.

- Screening Stage

The screening stage was further broken down into title and abstract screening and full text screening. The title and abstract screening were conducted for 205 articles. By reviewing the abstract and title, studies that were not relevant to our systematic literature review were excluded. Relevant studies were marked as included and those that required further review could be classified as ‘maybe’.

The screening of articles resulted in the elimination of 180 articles from the study, as those studies were not deemed significant to address the research objectives of this study. Twenty-five articles were further sought for retrieval of full text. However, two studies had to be further excluded due to non-availability of full text via university library sources and other public sources. A full text screening followed thereafter, and there were further exclusions of 11 studies owing to the content not being in English and due to non-suitability of the research.

**Figure 1:**
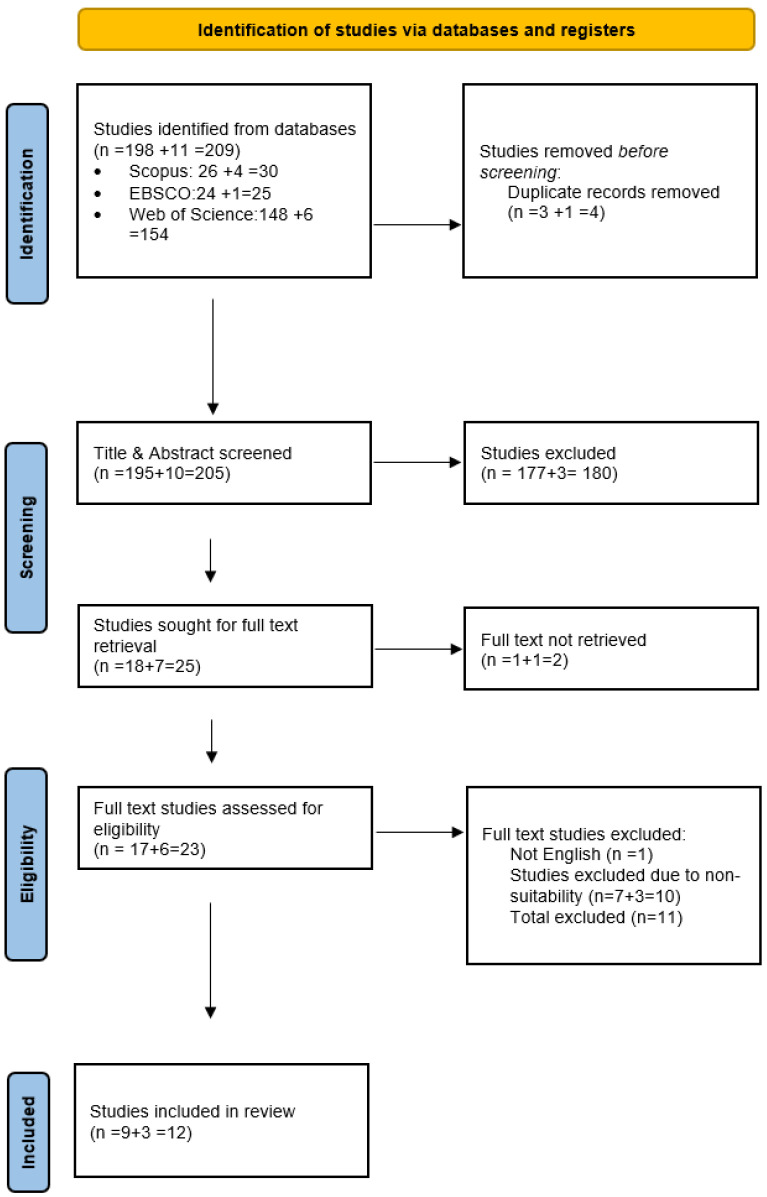
PRISMA Flow diagram for systematic reviews (Page et al., 2020, [46]).

Thus, a total of 12 studies have been included in the final list of included studies for this research. The PRISMA diagram for the study is provided in Figure 1.

#### Preliminary Insights

For the systematic literature review, a total of twelve studies have been included. Six studies out of twelve were included for evaluating Research Question 1. Four studies were included for Research Question 2, and one study was included in both the research questions. For Research Question 3, three studies were included. All included studies except one were academic papers from journals, while one was a conference paper. Regarding the research methods of the studies, five out of twelve employed quantitative analysis either through online surveys or questionnaires for the studies, and another five studies applied qualitative analysis techniques like interviews, ethnography, thematic analysis, etc. There were two studies which took a literature review approach, out of which one was a systematic literature review on smart technologies that were used during COVID-19, and the other took a multi-level review of the literature instead of the orthodox systematic review on the use of technologies within an existing smart city for building resilience during the pandemic. The below table (Table 3) is a representation of the studies in the systematic review.

### 3.2. Results and Analysis

In addressing Research Questions 1 and 2—specifically, the exploration of social media use during and after COVID-19 lockdowns and their contribution towards community pandemic resilience—we must clarify that the ‘lockdown period’ is not universally defined. Different countries enforced varying lockdown schedules; therefore, each study incorporated in this review adheres to the unique lockdown timelines of the countries investigated. We utilized the findings from these studies, cross-verifying their respective timeframes to categorize them under ‘lockdown’ and ‘post-lockdown’ periods. To enhance readability and coherence, we have organized the results according to themes correlating to the first two research questions. As for research Question 3—which aims to understand the role of smart cities in enhancing community resilience during the COVID-19 lockdown—the findings are presented separately.

Through reviewing of the included literature for this study, evident themes (both implicit and explicit) were identified for the lockdown and post-lockdown periods. The identified themes are connectedness, social support and wellbeing, information exchange, and resilience. The themes were identified based on the most prominent characteristics observed in the results of the studies. For the purposes of this study, the four themes can be described as follows:

*Connectedness*: In this study, the theme of ‘connectedness’ captures the various ways individuals used digital mediums to stay connected despite the physical distancing measures enforced during the pandemic. This included not just traditional methods of online communication, such as calls and messages, but also more interactive modes, like comments on social media platforms, tweets, and online music broadcasts. Essentially, any medium or characteristic that facilitated a sense of virtual closeness among individuals, enabling them to maintain their social relationships, fell under this thematic category.

*Social support and wellbeing*: This theme encompasses cases where individuals turned to each other or external resources to address their own or others’ emotional needs. It also includes situations where the support from social networks cultivated a sense of belonging among individuals.

*Information exchange*: During the pandemic, social media platforms were widely used as a source of information and a means for sharing information. The theme of information exchange is a broader classification for all medical information, general corona-virus-related information, information on restrictions or government regulations, or even information on preventive measures shared via social media (regardless of authenticity).

*Resilience*: Resilience is the outcome of the previous three themes in some studies, and in others it refers to the emotional and community resilience that was exhibited due to the presence of social media. To a certain extent, social media itself became a tool for building resilience in the community.

The results of the studies corresponding to each research question are detailed below:

#### 3.2.1. Research Question 1: How Was Social Media Used during COVID-19 Lockdown?

Social media has been studied extensively since it is so prominently used globally. The number of social media users worldwide in 2021 was nearly 4.2 billion (an increase from 3.6 billion in 2020), according to Datareportal [62]. This equates to more than 53% of the global population. Research on social media’s diverse applications spans across various domains, including mental health [63], crime [64], and marketing [65], among others. Nevertheless, there is a lack of comprehensive inquiry into social media’s role during the COVID-19 pandemic lockdown and its potential contribution to fostering community resilience. Therefore, by exploring the effectiveness of social media during lockdown, we aim to understand how social media was used during lockdown and whether it helped community resilience.

In this systematic literature review, out of the nine studies that explored social media usage, five of them specifically examined how social media was utilized during lockdown periods. Bukar et al. [51] examined both lockdown and post-lockdown periods. The studies were spread across multiple geographies, mainly Malaysia, Spain, Italy, France, the Netherlands, and from across the world. Table 4 provides the details of the studies included under this research question. Since the studies are spread across countries, they provide a broader picture—by considering studies from different geographical locations and that analyze various social media, the analysis can gain a range of perspectives, thereby enhancing the depth and breadth of the review. The social media platforms included in this study are WhatsApp, YouTube, Twitter, and TikTok. Noticeably, YouTube was the most widely studied in the literature.

Costa, Esteve-Del-Valle, and Hagedoorn [52] explored how WhatsApp reshaped interactions between people and enabled virtual proximity during the COVID-19 pandemic by conducting interviews and by examining content shared over WhatsApp, call details, etc. Fraser, Crooke, and Davidson [53] reported that online live-streamed music broadcasts delivered over YouTube facilitated social cohesion, thereby resulting in community resilience. Another social media platform that was studied was TikTok, which became one of the most downloaded social platforms during the initial wave of the pandemic, specifically during lockdown [54]. The study by Hiebert and Kortes-Miller [54] examined the online community on TikTok, which became a source of support for LGBTQ+ youth during the spring 2020 lockdown. Through a digital ethnography, human interactions on TikTok were examined, highlighting that during lockdown periods, these digital spaces replaced the temporarily inaccessible physical spaces and aided in the emotional and community resilience of LGBTQ+ youth while facing unprecedented trying situations. These three studies brought out similar theories and patterns of how using social media platforms during lockdown fostered connectedness, exchanging information, social support, and community resilience when faced with an adverse event as the COVID-19 pandemic.

Castaldo et al. [55] investigated community resilience through examining YouTube and Twitter big datasets focusing on French textual content. They observed a surge in online activities on both platforms during the lockdown period in France (March to May 2020), resulting from a decline in face-to-face interactions due to lockdown restrictions. Notably, this increase in online engagement and content consumption mostly took place at nighttime. Changes in the type of content being shared was also observed; the lockdown period saw an unforeseen drop in emotionally-driven content on both platforms, and there was a predictable shift from topics such as “social life” and “leisure” pre-lockdown, to themes related to “home” and “death” during the lockdown period. A study by van Leeuwen et al. [56] explored the use of social media during the lockdown period in the Netherlands, focusing specifically on leisure activities. Characterized as highly urbanized and densely populated, the Netherlands experienced a significant increase in “leisure-related” social media use during the lockdown between March and July 2020. The study also reports heightened stress and anxiety among its populace during this period of the pandemic. While van Leeuwen et al. [56] did not explicitly discuss community resilience, they highlighted the adaptability and resilience of the leisure sector. Specifically, they pointed to the innovative ways in which online digital platforms evolved to offer entertainment and home delivery services among other leisure-related services. This adaptability in business models suggests a broader community resilience, as the community found ways to navigate and adapt to the challenges of the lockdown through these digital transformations. A quantitative study on the Malaysian population was conducted by Bukar et al. [51] and also provided some insights on how crisis communication driven through social media platforms influences community resilience. They argue that community resilience is essential at a time of crisis in order to avoid panic and they therefore applied the situational crisis communication theory (SCCT), among other crisis communication and resilience models, to social media platforms during the pandemic. Briefly, 393 participants completed a questionnaire between July and November 2020. The findings show that during the lockdown periods in Malaysia, WhatsApp was the most popular social media platform, with 98.2% of respondents stating that they used it for communication throughout the lockdown period in Malaysia, followed by Facebook (82.1%), and then Instagram (72.7%), and then Twitter (57.1%). Several other platforms were also reportedly used, but at a much lower percentage. Bukar et al.’s [51] findings show that communication through social media platforms during the lockdown period was critical in building community resilience and overcoming the negative effects of lockdown. They view social media as a medium that has enabled citizens to become part of the crisis communication matrix.

Among the five studies that examined social media use during the lockdown period, various themes emerged regarding social media and resilience:

- Connectedness.
- Social Support and Wellbeing.
- Information Exchange.
- Emotional and Community Resilience.

These themes were prevalent in the studies discussing social media platforms during the various lockdown periods that different countries experienced, highlighting that these platforms have undoubtedly played an essential role during times of isolation. As various studies reveal, these platforms are not mere entertainment outlets; they serve as lifelines, facilitating emotional support, social connectivity, exchanging information, and creating a sense of belonging. Whether the close familial ties maintained via WhatsApp or the broader sense of community fostered by TikTok for marginalized groups, these digital spaces have emerged as a temporary replacement for the unattainable physical spaces that were pivotal for emotional wellbeing and community resilience during a time of collective crisis.

**Figure 2:**
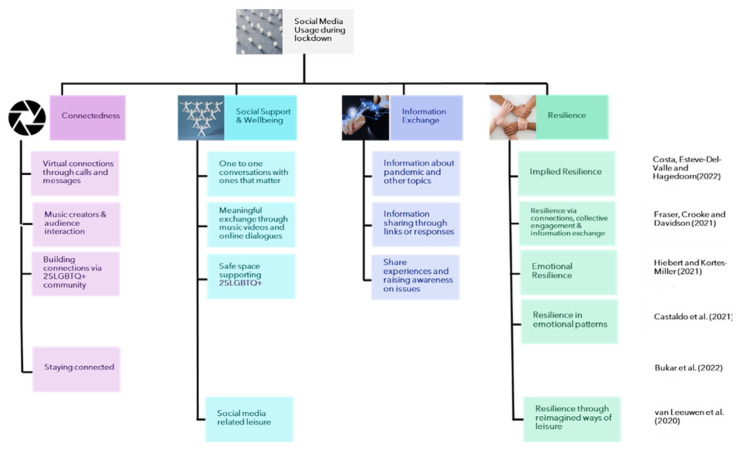
Thematic representation of social media usage during lockdown ([51,52,53,54,55,56]).

A classification of the themes is represented in Figure 2.

#### 3.2.2. Research Question 2: How Was Social Media Used after COVID-19 Lockdown in Contributing towards Pandemic Recovery and Community Resilience?

The aim of this research question was to investigate whether there were pandemic-induced changes in how individuals use social media post-lockdown. The studies that were included under this research question are geographically diverse and similar to lockdown-related studies, they explore a variety of social media platforms, such as Facebook, YouTube, Twitter, etc., thus providing a broad range of viewpoints. Table 5 is a representation of the studies included for this research question.

Saud, Mashud, and Ida [57] conducted a study in Indonesia during the Community Activities Restrictions Enforcement period, a time characterized by local or community-specific restrictions rather than a nationwide lockdown. The study, which applied a quantitative approach through an online survey, sought to understand the usage of various social media platforms (such as Facebook, YouTube, and Instagram; see Table 5 for a full list) and their role in facilitating information exchange and support. A significant finding was that a majority of the respondents viewed social media positively as a means of gathering medical information. Although the platforms under study were numerous, the authors did not specify which one primarily acted as a medium of connection. Nevertheless, they did note the overarching theme of connectedness; the respondents disclosed that they turned to social media to maintain ties with one another and the larger world during the pandemic. Additionally, Saud, Mashud, and Ida [57] reported that social media became a hub for sharing innovative activities to engage in, offering an avenue for emotional relief from the pandemic’s stress. This sentiment was further echoed by the observation that nearly 38.8% of the respondents perceived social media as a mechanism for social support, evidenced by the wishes and prayers users exchanged. While Saud, Mashud, and Ida [57] did not expressly identify resilience as a theme, it is reasonable to infer it, given the significant roles social media platforms played in fostering connectedness, sharing information, and social support, which are all factors that collectively contribute to community resilience during a crisis. However, it is important to note the study’s geographical specificity, limiting its broader applicability. As mentioned in the description of study for the previous research question, Bukar et al. [51] conducted a similar study based in Malaysia; however, the study extended over a period that covered both lockdown and post-lockdown periods, and the themes that emerged from their findings are similar to Saud, Mashud, and Ida’s [57].

Brailovskaia, Margraf, and Schneider [58] examined the relationship between social media usage (including platforms like Twitter and Facebook) and emotions during the COVID-19 pandemic across eight countries (see Table 5) via an online panel survey. They addressed the potential misinformation spread on social media platforms, suggesting that this could lead to increased stress among users. Other studies, such as Erku et al., [66] support the correlation between social media usage and stress. Ferguson et al. [67] found no compelling evidence linking social media or smartphone usage to individual mental wellbeing. It is worth noting that varying cultural factors or geographical contexts might influence these experiences, possibly leading to the contradicting findings observed in the studies.

Brailovskaia, Margraf, and Schneider [58] also highlighted that traditional media sources, like TV, remained more popular than online media in the eight countries they examined. However, there was a positive correlation observed between the psychological strain of COVID-19 and the use of social media platforms as an information source—the more psychological stress someone felt due to COVID-19, the more they turned to social media to obtain information. While the researchers point out the significance of social media in disseminating information, they also cautioned about the rapid spread of misinformation and fake news. They argued that responsible regulation of information shared on social media could potentially reduce the spread of COVID-19. Uran, Mohamed, and Aziz [59] also examined social media’s role in disseminating information; however, their focus was on university students in Malaysia. Their findings highlighted the students’ reliance on social media for updates from the government, connecting with family, friends, and lecturers, and entertainment. Additionally, the students utilized social media to support each other during challenging times, contributing to a richer learning experience. While Uran, Mohamed, and Aziz, [59] did not explicitly examine community resilience, it can be inferred from the observed support and awareness facilitated by social media. Interestingly, similar to the studies examining social media usage during lockdown, those in the post-lockdown phase also emphasized the prevalence of information sharing through these platforms.

**Figure 3:**
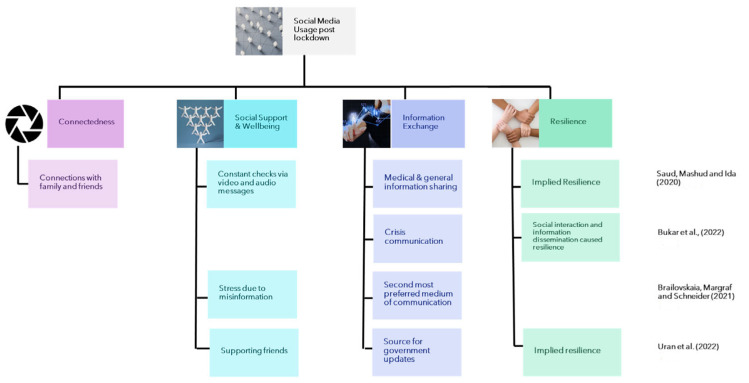
Thematic representation of social media usage post-lockdown ([51,57,58,59]).

The four studies examined for Research Question 2 also displayed similar themes to the previously examined studies. The themes are illustrated in Figure 3. It is evident that social media played role in fostering connectedness, facilitating information exchange, providing social support, and promoting wellbeing, even once lockdowns were no longer imposed on citizens. This indicates that the impact of social media on individuals and communities navigating pandemic-recovery is still ongoing and facilitating community resilience. While findings vary, influenced by cultural and geographical nuances, the consensus does promote the indispensability of social media as both a lifeline and a challenge. Responsible regulation and conscientious usage are paramount to maximizing benefits while mitigating potential harms.

#### 3.2.3. Research Question 3: Did Smart Technologies Play a Role in Community Resilience in Smart Cities during the COVID-19 Lockdown?

The aim of this research question was to understand the role of smart technologies, especially information and communication technologies (ICT), in urban spaces as they navigated the challenges of COVID-19. Furthermore, we aim to examine the interpretations of the “smart city” concept in the literature and the specific technologies that facilitated urban resilience during the pandemic (as shown in Table 6).

The concept of the smart city is evolving rapidly, with the pandemic triggering further discussions on its significance [37,68]. This surge in interest highlights the need to delve into historical perspectives on smart cities and understand the role of technology during crises.

Literature reviews reveal an increasing focus on smart cities, as illustrated by studies like dos Santos et al. [60]; Chu, Cheng, and Song [61]; and Sharifi, Khavarian-Garmsir, and Kummitha [37]. The World Bank [69] argues that resilient and sustainable cities were better pandemic managers, emphasizing the urgency for sustainability and smart city measures. Yet, the concept of the smart city is not without criticism; while Hollands [70] and Kitchin [38] challenge the idealized smart city image, Wong et al. [71] back its growth, referencing Chinese government initiatives.

Sassen and Kourtit [72] link urban migration to a city’s tech-driven resilience, suggesting that cities must digitize further to combat challenges such as pandemics, particularly when exacerbated in dense urban spaces. However, technological reliance brings vulnerabilities. Ijaz et al. [73] highlight the urgent need for increased security, particularly in pivotal sectors like healthcare and governance in smart cities. Meijer [44] further champions sustainable tech practices, arguing that the technological integration within cities is inevitable.

Dos Santos et al. [60] examined how ICT was instrumental in curtailing the pandemic’s spread. They outlined the integration of ICT across various city facets—infrastructure, communication, and intelligence—and the many strategies cities employed for resilience. The study describes a smart city as an evolving entity, merging new technology rooted in ICT with data-centric smart applications, offering tangible benefits to both humans and the environment. Their research highlighted innovative urban responses to COVID-19: employing robots and drones for sanitation and deliveries, implementing contact-reduction initiatives, and utilizing mobile apps for contact tracing and telemedicine. Additionally, Dos Santos et al. [60] highlighted the significant role of big data analytics and artificial intelligence in timely information dissemination to stakeholders. They argue that the adept integration and use of ICT played a crucial role in enhancing community resilience throughout the pandemic. The authors discuss the diverse and evolving nature of the smart city concept, defining it as the union of “new technology-based applications (based on ICT and data-driven smart applications) that enhance the human/environment interaction” ([60] p. 2). Importantly, their work did not restrict itself to a particular geographical area but broadly assessed strategies cities worldwide employed against COVID-19, reinforcing urban resilience.

Chu, Cheng, and Song [61] explored urban resilience in smart cities, particularly examining the impact of city size and governance capacity on resilience during the pandemic. Unlike dos Santos et al. [60], who detailed the technologies used in smart cities, Chu, Cheng, and Song [61] drew from real-time pandemic data across 276 Chinese cities. Their findings reflected the relationship between urban governance capacity and effective pandemic management, noting a 2.4% increase in recovered COVID-19 cases per capita for every unit increase of urban governance capacity. Although their study did not delve deeply into specific technologies, they advocated for the integration of advanced tools like big data and artificial intelligence in urbanization reforms. They concluded that smart city infrastructures bolstered pandemic management, suggesting an enhanced capacity for resilience against public crises. The overarching theme of their study highlighted the vital role of smart technologies in promoting sustainable urban development and resilience.

Sharifi, Khavarian-Garmsir, and Kummitha [37] conducted a systematic literature review examining smart cities and how their solutions facilitated resilience during the pandemic. They recognized, similar to dos Santos et al. [60], that while smart cities often emphasize “ICT-enabled technologies”, there is an emerging dimension encompassing non-physical aspects such as institutions and knowledge economy, which intermingle with physical infrastructure. These authors point out that the smart city concept will continue to evolve with technology. Their review offers an insightful examination of technology’s role throughout pandemic stages, categorizing resilience into planning, absorption, recovery, and adaptation, and assessing tech’s role in each. This comprehensive study highlights technology’s cruciality in enhancing city resilience.

Similar to Chu, Cheng, and Song [61], Sharifi, Khavarian-Garmsir, and Kummitha [37] argue for the need for more investments in smart technologies, noting their potential in early crisis detection and tracking. They also emphasize the importance of smart city discussions post-pandemic, suggesting these initiatives can equip cities to handle future challenges better and aid in pandemic recovery and resilience.

To sum up, while the importance of technology in smart cities is extensively discussed by dos Santos et al. [60] and Sharifi et al. [37], Chu, Cheng, and Song [61] spotlight the significance of sustainable city development, suggesting a heavier emphasis on technologies like AI and big data.

## 4. Conclusions, Limitations, and Future Scope

The primary focus of this systematic literature review was to examine the role of social media during both the lockdown and post-lockdown phases of the pandemic and its facilitation of community resilience. The study also aimed to understand “smart cities” and assess the contribution of smart technologies to resilience within these cities.

There were evident consistent patterns of social media usage during the lockdown and post-lockdown periods. Themes of social support and information exchange were prevalent in discussions for both phases, highlighting the community’s reliance on social media for both emotional backing and crucial information in order to facilitate community resilience. The data from the studies suggest a positive correlation between community resilience and social media usage, a conclusion drawn from the predominantly beneficial outcomes associated with social media use found in the studies.

Our third research question examined smart cities. While many studies delved into the definition and benefits of smart cities for resilience, there was an absence of discussions centered on the sustainability of these technologies. This gap suggests that the focus might lean more towards immediate technological solutions and their benefits, with less emphasis on their long-term sustainability, especially in the context of unforeseen events such as pandemics.

Based on the outcome of this systematic literature review, we recommend that given the positive correlation between community resilience and social media usage, policymakers could invest in training and strategies that leverage social media platforms for crisis management. This could include creating official channels to disseminate crucial information, mobilize support, and counter misinformation. We also recommend more direct ways of engagement, such as, for example, the development of a smartphone app aimed at enabling users to ‘map’ their resilience and stress levels, which could empower citizens and bridge the gap between social media’s influence on urban community resilience and the role of sustainable smart cities. Understanding how these two domains intersect could pave the way for more holistic resilience strategies. With user permission, these data can be anonymously aggregated to provide a ‘heat map’ of community resilience and stress. Areas with lower resilience or higher stress could be quickly identified, allowing for targeted community support, resources, or interventions. Such an app can provide real-time feedback directly to policymakers or local leaders, sharing specific concerns or suggestions. By offering a direct, actionable, and user-friendly means of gauging and enhancing community resilience, we can reimagine crisis management and post-crisis recovery. Harnessing such user-generated data would not only empower individuals but could provide invaluable data to those in positions to make community-wide decisions, not only aiding in future crisis prevention and/or management but also facilitating a speedy recovery to help ensure that our communities have the tools to remain resilient.

This systematic literature review has offered a deeper examination of the manner in which community resilience was fostered through the utilization of social media during distinct phases of the pandemic—the lockdown and post-lockdown periods. It also examined the role smart technologies play in fortifying this resilience. However, like any research, this review is not exempt from certain limitations. The limited number of studies that particularly addressed urban community resilience in the context of social media usage and sustainable smart cities compelled the review to broaden its scope, focusing on community resilience more generally. Given the short timeframe of this review (the lockdown periods and the months that immediately followed), a longitudinal analysis of social media’s impact on community resilience during different phases of crises would offer comprehensive insights into its long-term effects. Subsequent research could focus on specific geographical or cultural contexts to understand any regional differences in community resilience via social media and smart cities.

## Figures and Tables

**Table 1:** List of search terms harvested using PICO framework for Research Question 1 and 2.

Population	Intervention	Comparison	Outcome
**Community**	social media	during lockdown	community resilience
**Society**	social platforms	in lockdown	urban resilience
**Neighborhood**	social networking websites	post lockdown	neighborhood resilience
**Neighbourhood**	social media website	after lockdown	pandemic recovery
**Communities**	social media service	Shutdown	pandemic response
**Urban communities**	social networks	Curfew	positive adaptation
**Urban environment**	social networking services	Restrictions	COVID-19 response
	SNS	COVID-19 lockdown	COVID-19 recovery
	Twitter	Quarantine	coronavirus response
	Facebook	border closure	recover/recovery
	Instagram	isolation/isolate	respond/response
	Pinterest	stay-at-home	resilience
	YouTube	Solitary	withstand/withstand adversity
	Linkedin	Quarantine	adapting/adapt
	Tiktok	Confinement	adaptive behaviour/behavior
	Snapchat	detention/detain	social media usage
	Whatsapp	separate/separation	
	Wechat	seclude/secluded	
	Tumblr	ostracize/ostracise	
	Reddit		
	Telegram		

**Table 2:** List of search terms harvested using PICO framework for Research Question 3.

Population	Intervention	Comparison	Outcome
**smart cities**	smart technologies	during lockdown	community resilience
**digital city**	smart infrastructure	in lockdown	urban resilience
**connected cities**	smart technology	post lockdown	neighborhood/neighbourhood resilience
**smart cities mission**	smart living	after lockdown	pandemic recovery
**smart city**	smart mobility	shutdown	pandemic response
**information city**	artificial intelligence	curfew	positive adaptation
**knowledge-based city**	smart governance	restrictions	COVID-19 response
**ubiquitous city**	ICTs	COVID-19 lockdown	COVID-19 recovery
**wired city**	information and communications technology	quarantine	coronavirus response
**intelligent city**		border closure	recover/recovery
**Busan**		isolation/isolate	respond/response
**London**		stay-at-home	resilience
**Santander**		solitary	withstand/withstand adversity
**sustainable smart cities**		quarantine	adapting/adapt
		confinement	adaptive behaviour
		detention/detain	
		separate/separation	
		COVID-19	
		ostracize/ostracise	

**Table 3:** List of included studies for the systematic review ([17,51,52,53,54,55,56,57,58,59,60,61]).

No	Reference	Research Question	Type	Technique
1	Bukar et al. (2022) [51]	1 & 2	Academic paper	Quantitative study through online questionnaire with 393 responses
2	Castaldo et al. (2021) [55]	1	Academic paper	Emotional & thematic analysis of 100 thousand YouTube videos and a collection of 8 million tweets
3	Costa, Esteve-Del-Valle and Hagedoorn (2022) [52]	1	Academic paper	30 semi-structured, in-depth interviews, 10 in each city
4	Fraser, Crooke and Davidson (2021) [53]	1	Academic paper	Online ethnographic approach for filtering music content (10 case studies spread across geographically)
5	Hiebert and Kortes-Miller (2021) [54]	1	Academic paper	Digital ethnography and thematic analysis
6	Van Leeuwen et al. (2020) [56]	1	Academic paper	Qualitative analysis
7	Brailovskaia, Margraf and Schneider (2021) [58]	2	Academic paper	Population based online panel survey of 8302 participants
8	Saud, Mashud and Ida (2020) [57]	2	Academic paper	Quantitative research through online survey with 348 responses
9	Uran et al. (2022) [59]	2	Academic paper	Qualitative research through in-depth interviews of 20 University students
10	Chu, Cheng and Song (2021) [61]	3	Academic paper	Quantitative analysis
11	dos Santos et al. (2021) [60]	3	Conference paper	Literature review
12	Sharifi, Khavarian-Garmsir and Kummitha (2021) [17]	3	Academic paper	Systematic literature review of 147 studies

**Table 4:** List of included studies for Research Question 1 ([51,52,53,54,55,56]).

No	Reference	Time Frame	Lockdown Status	Geography	Social Media
1	Costa, Esteve-Del-Valle and Hagedoorn (2022) [52]	March and April 2020	Lockdown	Barcelona (Spain), Milan (Italy), and Groningen (the Netherlands)	WhatsApp
2	Fraser, Crooke and Davidson (2021) [53]	1st April to 30th October 2020	Lockdown	Worldwide	YouTube
3	Hiebert and Kortes-Miller (2021) [54]	1st April 2020 to 30th June 2020	Lockdown	Unspecified	TikTok
4	Castaldo et al. (2021) [55]	17th February and 14th April 2020	Lockdown	France	Twitter and YouTube
5	Bukar et al. (2022) [51]	July 2020 to November 2020	Lockdown and post lockdown	Malaysia	Facebook, Twitter, Snapchat, Skype, WeChat, TikTok, LinkedIn, Telegram, Weibo, Tumblr, Quora, Viber, YouTube, Instagram, WhatsApp, QQ, Line, Reddit, Pinterest, Zoom and Imo
6	Van Leeuwen et al. (2020) [56]	Unspecified	Intelligent lockdown	Netherlands	Unspecified

**Table 5:** List of included studies for Research Question 2 ([51,57,58,59]).

No	Reference	Time Frame	Lockdown Status	Geography	Social Media
1	Saud, Mashud and Ida (2020) [57]	March 2020 to April 2020	“Community activities restrictions enforcement”	Indonesia	Facebook, YouTube, Instagram, WhatsApp and Line
2	Bukar et al. (2022) [51]	July 2020 to November 2020	During the pandemic	Malaysia	Facebook, Twitter, Snapchat, Skype, WeChat, TikTok, LinkedIn, Telegram, Weibo, Tumblr, Quora, Viber, YouTube, Instagram, WhatsApp, QQ, Line, Reddit, Pinterest, Zoom and Imo
3	Brailovskaia, Margraf and Schneider (2021) [58]	End of May to beginning of June 2020	During the pandemic	Unspecified	Facebook, Twitter etc.
4	Uran et al. (2022) [59]	Unspecified	Lockdown (mention of social media during lockdown, hence assumed)	Malaysia	Unspecified

**Table 6:** List of included studies for Research Question 3 ([17,60,61]).

No	Reference	Time Frame	Geography
1	Dos Santos et al. (2021) [60]	Pandemic	Unspecified
2	Chu, Cheng and Song (2021) [61]	Pandemic	China
3	Sharifi, Khavarian-Garmsir and Kummitha (2021) [17]	Pandemic	Unspecified

## Data Availability

Data sharing is not applicable to this article—no new data was created or collected in this study.
